# Mechanism and regulation of sorbicillin biosynthesis by *Penicillium chrysogenum*

**DOI:** 10.1111/1751-7915.12736

**Published:** 2017-06-15

**Authors:** Fernando Guzmán‐Chávez, Oleksandr Salo, Yvonne Nygård, Peter P. Lankhorst, Roel A. L. Bovenberg, Arnold J. M. Driessen

**Affiliations:** ^1^ Molecular MicrobiologyGroningen Biomolecular Sciences and Biotechnology InstituteUniversity of GroningenNijenborgh 7, 9747 AG GroningenThe Netherlands; ^2^ Synthetic Biology and Cell EngineeringGroningen Biomolecular Sciences and Biotechnology InstituteUniversity of GroningenNijenborgh 7, 9747 AG GroningenThe Netherlands; ^3^ DSM Biotechnology CenterAlexander Fleminglaan 12613 AX DelftThe Netherlands; ^4^ Present address: Biology and Biological EngineeringIndustrial BiotechnologyChalmers University of TechnologyKemigarden 4 GöteborgSweden

## Abstract

*Penicillium chrysogenum* is a filamentous fungus that is used to produce β‐lactams at an industrial scale. At an early stage of classical strain improvement, the ability to produce the yellow‐coloured sorbicillinoids was lost through mutation. Sorbicillinoids are highly bioactive of great pharmaceutical interest. By repair of a critical mutation in one of the two polyketide synthases in an industrial *P. chrysogenum* strain, sorbicillinoid production was restored at high levels. Using this strain, the sorbicillin biosynthesis pathway was elucidated through gene deletion, overexpression and metabolite profiling. The polyketide synthase enzymes SorA and SorB are required to generate the key intermediates sorbicillin and dihydrosorbicillin, which are subsequently converted to (dihydro)sorbillinol by the FAD‐dependent monooxygenase SorC and into the final product oxosorbicillinol by the oxidoreductase SorD. Deletion of either of the two *pks* genes not only impacted the overall production but also strongly reduce the expression of the pathway genes. Expression is regulated through the interplay of two transcriptional regulators: SorR1 and SorR2. SorR1 acts as a transcriptional activator, while SorR2 controls the expression of *sorR1*. Furthermore, the sorbicillinoid pathway is regulated through a novel autoinduction mechanism where sorbicillinoids activate transcription.

## Introduction

Sorbicillinoids are a large family of hexaketide metabolites that include more than 90 highly oxygenated molecules. These compounds can be structurally classified into four groups: monomeric sorbicillinoids, bisorbicillinoids, trisorbicillinoids and hybrid sorbicillinoids (Meng *et al*., 2016). Sorbicillinoids were originally isolated from *Penicillium notatum* in 1948, but found later also in the culture broths of marine and terrestrial ascomycetes (Harned and Volp, 2011). In particular, *P. chrysogenum* strain NRRL1951 has been reported to be a natural source of more than 10 sorbicillinoids (Meng *et al*., 2016). This fungus was the progenitor for the high‐β‐lactam‐yielding strains that are currently used in industry. These strains were obtained by several decades of classical strain improvement, where an early goal was to eliminate the production of yellow pigments as contaminants of β‐lactams. This resulted in the loss of sorbicillinoid production through mutagenesis of a key polyketide synthase gene (Salo *et al*., 2015). Recently, the interest in sorbicillinoids was revived because of the wide bioactivity spectrum associated with these molecules and their potential pharmaceutical value. For instance, sorbicathecols A/B inhibits the cytopathic effect induced by HIV‐1 and influenza virus A (H1N1) in MDCK cells (Nicoletti and Trincone, 2016), whereas isobisvertinol inhibits lipid droplet accumulation in macrophages, an event associated with the initiation of atherosclerosis (Koyama *et al*., 2007; Xu *et al*., 2016). Moreover, the oxidized form of bisvertinol, bisvertinolone, displays a potent cytotoxic effect against HL‐60 cells and is an antifungal via inhibition of β(1,6)‐glucan biosynthesis (Nicolaou *et al*., 2000; Du *et al*., 2009). Other sorbicillinoids, such as oxosorbicillinol and dihydrosorbicillinol, were shown to exhibit antimicrobial activity against *Staphylococcus aureus* and *Bacillus subtilis* (Maskey *et al*., 2005).

Despite the wide spectrum of bioactive properties reported for sorbicillinoids, the biosynthetic pathway of these polyketides has not yet been elucidated. Isotope labelling studies suggested that the hexaketide structure of sorbicillinol is assembled by a Claisen‐type reaction involved in carbon–carbon bond formation (Sugaya *et al*., 2008; Harned and Volp, 2011), whereas Diels–Alder‐ and Michael‐type reactions have been proposed as the most probable mechanism for the formation of sorbicillinoid dimers (Maskey *et al*., 2005; Du *et al*., 2009). Recently, two polyketide synthases (PKS) have been implicated in the biosynthesis of sorbicillactone A/B in *P. chrysogenum* E01‐10/3. The presumed PKS genes belong to a gene cluster that comprises five additional open reading frames (ORFs) (Avramović, 2011). Commonly, PKS enzymes form the scaffold structure of a molecule that is then further modified by tailoring enzymes, often encoded by genes localized in the vicinity of the key PKS genes (Lim *et al*., 2012). Indeed, a FAD‐dependent monooxygenase has been identified as part of the putative sorbicillin cluster, and this enzyme was shown to convert (2′,3′‐dihydro)sorbicillin into (2′,3′‐dihydro)sorbicillinol (Fahad *et al*., 2014).

The putative sorbicillinoid gene cluster of industrial *P. chrysogenum* strains includes a highly reducing PKS (*sorA*,* Pc21 g05080*) and a non‐reducing PKS (*sorB, Pc21 g05070*) (Salo *et al*., 2016). The *sorA* gene was shown to be essential for sorbicillinoid biosynthesis, as its deletion abolishes the production of all related compounds (Salo *et al*., 2015, 2016). In addition, this cluster harbours five further genes, two genes encoding putative transcription factors (*sorR1* and s*orR2, Pc21 g05050* and *Pc21 g05090*, respectively), a transporter protein (*sorT, Pc21 g05100*), a monooxygenase (s*orC, Pc21 g05060*) and an oxidase (*sorD, Pc21 g05110*). A recent study in *Trichoderma reesei* indicates that homologous transcriptional factors are involved in the regulation of sorbicillinoid biosynthesis in this fungus (Derntl *et al*., 2016), but the exact mechanism of regulation remained obscure. Here, we have resolved the biosynthetic pathway of sorbicillinoid biosynthesis and its regulation by metabolic and expression profiling of individual gene knockout mutants. The data show that SorR1 is a transcriptional activator, whose expression is controlled by the second regulator SorR2. Furthermore, transcription is regulated through an autoinduction mechanism by sorbicillinoids, the products of the pathway.

## Results

### Metabolic profiling of strains with individual deletions of the sorbicillinoid biosynthesis genes

**Figure 1:**
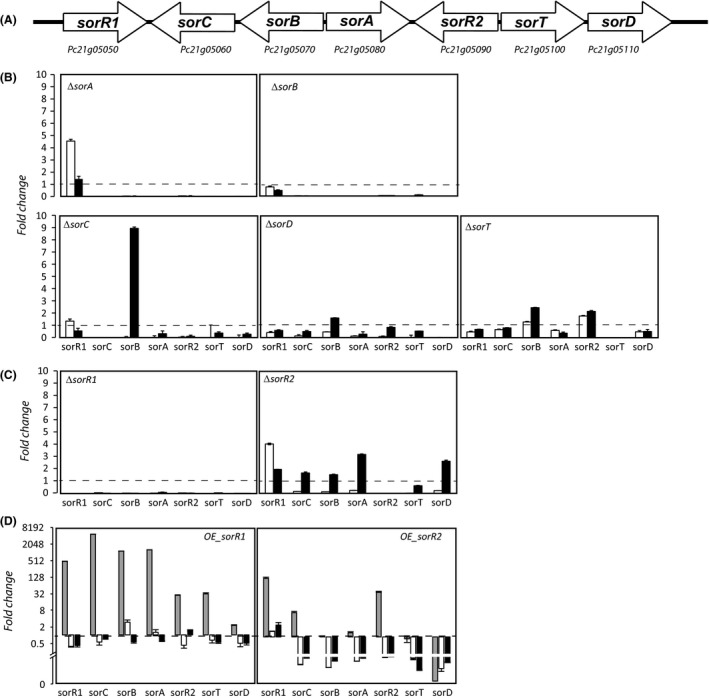
Relative expression of the genes of the sorbicillinoid biosynthesis gene cluster. A. Schematic representation of the gene cluster: *Pc21 g05050 (sorR1; transcriptional factor), Pc21 g05060 (sorC; monooxygenase), Pc21 g05070 (sorB*; non‐reduced polyketide synthase*), Pc21 g05080 (sorA*; highly reduced polyketide synthase*), Pc21 g05090 (sorR2*; transcriptional factor*), Pc21 g05100 (sorT*; MFS transporter*) and Pc21 g05110 (sorD*; oxidase*)*. B. Quantitative real‐time PCR analysis in sorbicillinoid gene cluster expression in strains with individual deleted *sorA*,* sorB*,* sorC*,* sorD* and *sorT*. C. *sorR1* and *sorR2* genes. D. qPCR analysis in strains and overexpressed *sorR1* and *sorR2*. Samples were taken at day 1 (grey bars), 3 (white bars) and 5 (black bars). Data shown as fold change relative to *P. chrysogenum* DS68530Res13 strain.

*Penicillium chrysogenum* strain DS68530Res13 produces high levels of sorbicillinoids causing yellow pigmentation of the culture broth. This strain is derived from strain DS68530 as described previously (Salo *et al*., 2016). The proposed sorbicillin biosynthetic gene cluster (Fig. 1A) includes the previously characterized polyketide synthase gene *sorA* (*Pc21 g05080*), a second polyketide synthase (Pc21 g05070, *sorB*), two transcriptional factors (*Pc21 g05050, sorR1*;* Pc21 g05090*,* sorR2*), a transporter protein (*Pc21 g05100*,* sorT*), a monooxygenase (Pc21 g05060, *sorC*) and an oxidoreductase (Pc21 g05110, *sorD*). These genes were individually deleted, and metabolic profiling was performed on the supernatant fractions of cultures of the respective strains. The metabolic profiling searched for the previously identified sorbicillinoid‐related compounds as well as potential new molecules. Furthermore, the expression of the aforementioned genes was analysed by qPCR to exclude possible polar effects of the gene deletions and to assess the impact of the deletion of the two putative regulators on the expression of the sorbicillinoid gene cluster.

**Figure 2:**
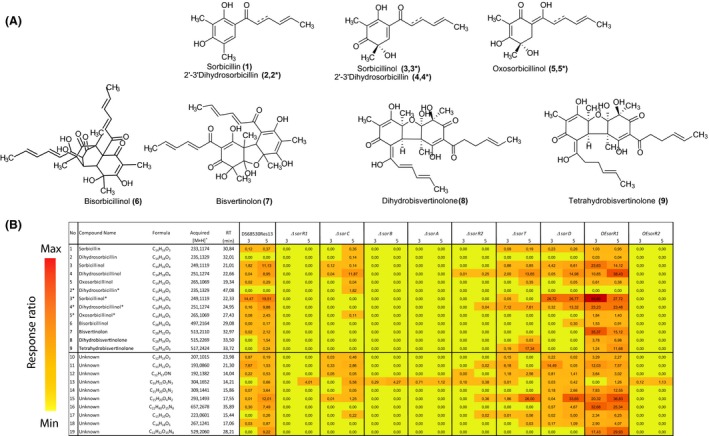
A. Sorbicillinoid‐related compounds detected in this study. B. Response ratio of the sorbicillinoid concentrations in the culture broth of indicated sorbicillin‐producing *P. chrysogenum* strains. Reserpine was used as internal standard for normalization. Compounds were detected after 3 and 5 days of growth. The mass‐to‐charge ratio (*m/z*) of the protonated metabolites, their empirical formulas and retention time (RT) are indicated. Structures of sorbicillin‐related compounds detected in this study. (*) Indicates an isomer of the known sorbicillinoids.

In the *ΔsorA* mutant which lacks the highly reducing polyketide synthase, no sorbicillinoids could be detected in the culture supernatant (Fig. 2B) confirming our earlier observations (Salo *et al*., 2016). Also in the *ΔsorB* mutant, which lacks the non‐reducing polyketide synthase, sorbicillinoid production was completely abolished (Fig. 2B). These data are consistent with the notion that SorA and SorB are responsible for the formation of the core (dihydro‐)sorbicillin structure. The unknown compound [13] was present at elevated levels in both the *ΔsorA* and *ΔsorB* mutants as compared to the parental strain and thus is most likely not related to sorbicillins. This compound has a retention time (RT) of 14.21 min, *m/z* [H]^+^ of 304.1652 and a calculated empirical formula C_16_H_21_O_3_N_3_ (Fig. 2B). All the other unknown compounds listed in Fig. 2 appear to be associated with the sorbicillinoid biosynthetic pathway. Importantly, in both the *ΔsorA* and in *ΔsorB* mutants, none of the cluster genes except the *sorR1* gene were expressed (Fig. 1B).

Next, the role of the individual genes encoding the enzymes of the pathway was analysed. Deletion of the monooxygenase gene *sorC* resulted in a 1.3 times increase in dihydrosorbicillinol [4]. The *ΔsorC* mutant showed lower levels of sorbicillin and sorbicillinol, which is consistent with the proposed role of SorC protein in the oxidative dearomatization of sorbicillin [1] into sorbicillinol [3*] (Fahad *et al*., 2014). In this strain, we also noted an upregulation of the *sorB* gene and the partial downregulation of the rest of the cluster (Fig. 1B). The main compound produced after 3 days in the *ΔsorD* strain, which lacks the putative oxidoreductase, was sorbicillinol [3;3*]. After 5 days, elevated levels of compound [15] with the m/z [H]^+^ of 293.1493 were also noted. Oxosorbicillinol [5*] with an empirical formula of C_14_H_16_O_5_ and m/z [H]^+^ of 265.1069 was not detected in this strain, which suggests that SorD is involved in the conversion of sorbicillinol into oxosorbicillinol. The *ΔsorD* strain showed a slight overexpression (0.6 times higher) of the *sorB* gene while the other genes of the pathway were about twofold downregulated (Fig. 1B). The *ΔsorT* mutant which lacks the putative transporter showed a similar gene expression as the parental strain with only minor changes in *sorB* and *sorR2* expression. In this strain, the production of sorbicillinoids shifted mostly towards tetrahydrobisvertinolone [9] and the compound with an empirical formula C_15_H_20_O_4_N_2_ [15].

Summarizing, our data suggest that the monooxygenase SorC is involved in the oxidative dearomatization of sorbicillin [1] into sorbicillinol [3*] and that the oxidoreductase SorD converts sorbicillinol [3;3*] into oxosorbicillinol [5*]. No clear role can be attributed to the transporter SorT. Furthermore, the individual deletion of the biosynthesis genes also impacts the regulation of the pathway.

### Deletion and overexpression of the regulatory genes sorR1 and sorR2

The sorbicillinoid biosynthetic gene cluster contains two genes encoding putative regulators, i.e. *sorR1* and *sorR2*. The deletion of *sorR1* abolished the expression of the entire sorbicillin biosynthesis gene cluster, and consequently, all sorbicillinoid‐related compounds were absent in this strain (Figs 1C and 2B). The deletion of *sorR2* impacted the expression after 3 days (Fig. 1C), while after 5 days, the expression profiles were equal or even higher than in the parental strain (Fig. 1C). Intriguingly, despite the biosynthetic genes being expressed, hardly any sorbicillinoids were present in the culture broth of the *ΔsorR2* strain, except for very low levels of dihydrosorbicillinol [4] (Fig. 2B). These data suggested that SorR1 is essential for the regulation of sorbicillinoid biosynthesis, whereas the absence of SorR2 results in a delayed expression of the pathway genes.

Overexpression of *sorR1* (*OEsorR1*) or *sorR2* (*OEsorR2*) resulted in elevated levels of these regulators in the early stages of the cultivation (Fig. 1D). In the *OEsorR1* strain, this also substantially elevated the expression of the other pathway genes which suggests that SorR1 acts as a transcriptional activator. Concomitantly, the overexpression of *sorR1* massively increased the sorbicillinoid production (Fig. 2B). In contrast, in the OEsorR2 mutant, the expression of nearly all the genes of the sorbicillinoid cluster was strongly reduced, except for *sorR1* expression which was increased. Consequently, production of all sorbicillinoid‐related compounds was reduced. These data suggest that SorR2 is involved in a complex mechanism of regulation and likely acts in concert with SorR1 which is a transcriptional activator of the pathway.

### Sorbicillinoids activate gene expression

**Figure 3:**
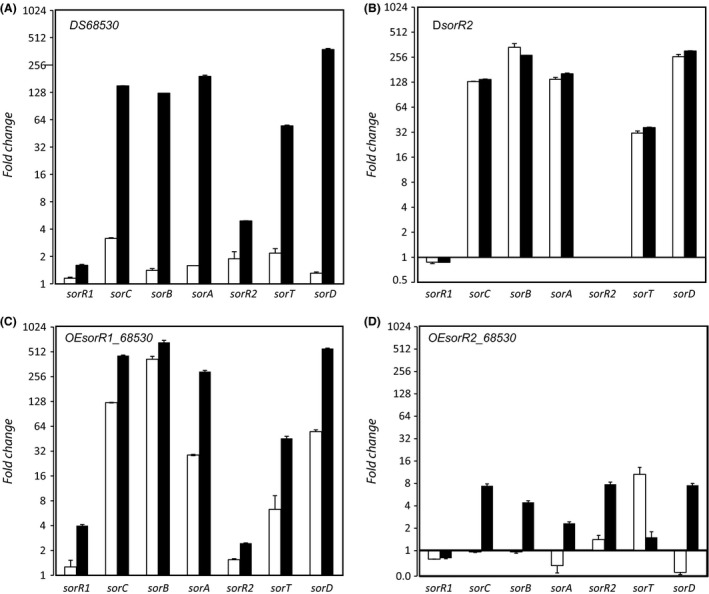
Relative expression of the sorbicillinoid cluster genes in the presence (black bars) and absence (white bars) of sorbicillinoids in the growth medium. Strains: A. DS68530, B. *ΔsorR2*, C. *OEsorR1*_68530 and D. *OEsorR2_*68530. Samples were taken after 3 days of growth. Data shown as fold change relative to *P. chrysogenum* DS68530 strain.

The observation that deletion of the PKS enzymes SorA and SorB, and consequently a loss in sorbicillinoid production, causes a marked reduction in the expression levels of the biosynthesis genes suggests that sorbicillinoids influence the expression of the pathway through an autoinduction regulatory process. To test this hypothesis, a culture of strain DS68530, which itself does not produce sorbicillinoids because of the mutation in SorA, was fed with a sorbicillinoid containing spent medium derived from the DS68530Res13 strain. This resulted in highly increased expression of all sorbicillinoid biosynthetic genes (Fig. 3A), except for the two regulatory genes, the expression of which remained unchanged. As a control, the cells were fed with supernatant derived from the non‐sorbicillin‐producing strain DS68530, and this had no impact on the expression of the sorbicillinoid gene cluster. These data suggest that the sorbicillinoid biosynthetic gene cluster is regulated through an autoinduction mechanism by which the products of the pathway, the sorbicillinoids, stimulate the expression of the pathway genes.

To examine the autoinduction mechanism in greater detail, the effect of sorbicillinoid addition was also tested for the *ΔsorR1* and *ΔsorR2* strains, and strains overproducing *sorR1* (*OEsorR1*) or *sorR2* (*OEsorR2*) in the genetic background of the non‐sorbicillin‐producing strain DS68530. Expression of the cluster genes remained unaffected in the *ΔsorR1* and *ΔsorR2* strains when cells were grown in the presence of sorbicillinoids (data not shown, Fig. 3B). Overproduction of *sorR1* resulted in the elevated expression of the pathway genes which was further stimulated by the presence of sorbicillinoids in the culture medium (Fig. 3C). A similar result was obtained with the overexpression of *sorR2*, albeit the effect of sorbicillinoids was at least two orders of magnitude lower (Fig. 3D). It should be noted that *sorT* in the *OEsorR2* strain was highly overexpressed. This gene lies downstream of *sorR2,* and due to strain construction, it is no longer expressed from its endogenous promoter but controlled by the strong *gndA* promoter (Polli *et al*., 2016). Taken together, these data suggest that sorbicillinoids autoinduce the sorbicillinoid biosynthetic pathway in a process that requires the combined activity of the transcriptional regulators SorR1 and SorR2.

## Discussion

**Figure 4:**
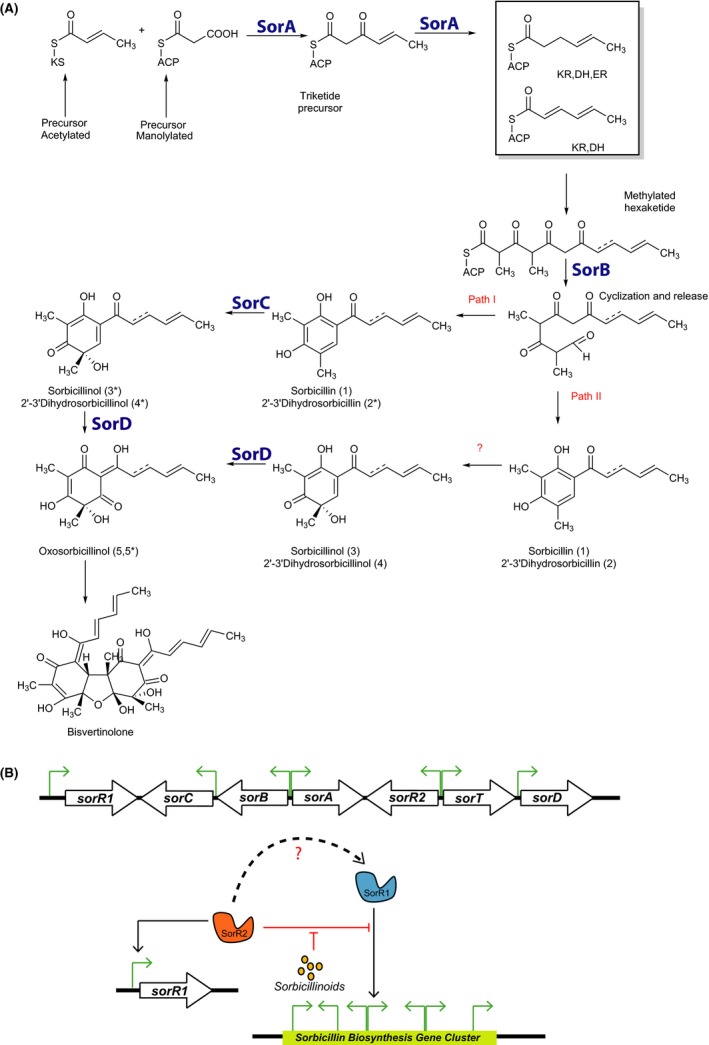
Proposed model of the sorbicillin biosynthetic pathway and its regulation. A. PKS domains in SorA and SorB are abbreviated as KS (ketosynthase), ACP (acyl carrier protein), KR (ketoreductase), DH (dehydratase), MT (methyltransferase), ER (enoylreductase). Adapted from Ref. (Avramović, 2011; Fahad *et al*., 2014; Derntl *et al*., 2016; Salo *et al*., 2016). B. The autoinduction mechanism of pathway gene expression by sorbicillinoids which involves the two transcriptional factors SorR1 and SorR2. On top, a schematic representation of the gene cluster. Black solid arrows indicate a positive stimulation and the red arrows show a negative effect. Green arrows represent the promoters of the indicated genes.

*Penicillium chrysogenum* produces large amounts of sorbicillinoids. In a previous study, we have identified one of the polyketide synthases (SorA) involved in this process (Salo *et al*., 2016). To resolve the biosynthetic mechanism of sorbicillinoid production, each of the genes of the putative cluster was individually deleted and analysed by metabolic profiling. Our data indicate that the two polyketide synthase genes, *sorA* and *sorB,* are both required for sorbicillinoid production. Our metabolic profile analysis did not reveal possible intermediate products of the polyketide synthases. However, it has previously been suggested that these two proteins are responsible for the synthesis of the basic hexaketide scaffold (Fig. 4A) (Harned and Volp, 2011; Fahad, 2014). Biosynthesis of sorbicillin or dihydrosorbicillin depends on the functionality of the enoylreductase (ER) domain of SorA, while the methylation of the hexaketide derived from SorA, prior to the cyclization, is catalysed by SorB. Our deletion analysis further suggests that SorC, a monooxygenase, is needed for the conversion of dihydrosorbicillin [2*] and sorbicillin [1] into dihydrosorbicillinol [4*] and sorbicillinol [3*], respectively, which confirms previous observations on the biochemical characterization of this enzyme (Fahad *et al*., 2014). Nevertheless, SorC is apparently not the only enzyme or mechanism involved in this conversion step as low amounts of likely tautomer forms of (dihydro)sorbicillinol [4;3] (Harned and Volp, 2011) were still detected in the supernatant of the *ΔsorC* mutant. In the *ΔsorD* strain, sorbicillinol [3;3*] accumulated while oxosorbicillinol [5*] could not be detected. This suggested that SorD is an oxidase that converts sorbicillinol into oxosorbicillinol [5*], which is also a stable compound. Although low amounts of the potential tautomer [5] have been previously detected (Maskey *et al*., 2005), this molecule might be the result of the spontaneous oxidation of sorbicillin (Bringmann *et al*., 2005). Furthermore, we could not detect dihydrobisvertinolone [8] and tetrahydrobisvertinolone [9] in the supernatant, which is in line with the proposed function of SorD as the product oxosorbicillinol is a precursor for bisvertinolone synthesis (Abe *et al*., 2002). Deletion of the gene specifying the transporter SorT only marginally affected sorbicillinoid production, and thus, no clear transport function could be assigned to this protein. Summarizing, the functional assignment of the various gene products resulted in a biosynthetic scheme depicted in Fig. 4. A similar pathway was recently constructed for *Trichoderma reesei* (Astrid R. Mach‐Aigner, pers. comm.).

To understand the mechanism of regulation of the pathway, we analysed the effect of the deletion and overexpression of the two putative transcriptional regulators, *sorR1* and *sorR2,* that are part of the gene cluster. The data indicate that SorR1 is needed for the transcriptional activation of sorbicillinoid gene cluster. In the *sorR1* deletion strain, the expression of all biosynthetic genes was completely abolished and consequently, sorbicillinoid production was eliminated. In the overexpression strain, cluster genes were upregulated causing an earlier onset of sorbicillinoid biosynthesis. *SorR2* appears to fulfil a more complex role. Deletion of *sorR2* caused a later onset of the expression of the sorbicillinoid genes, which explains the low amounts of sorbicillinoids that are still detected in that strain. In the *sorR2* overexpression strain, the transcriptional levels of *sorR1* were strongly enhanced, while the expression of the pathway genes was strongly reduced at later stages of growth. Moreover, *sorD* was not expressed and overall sorbicillinoid production was abolished. This observation suggests a complex mechanism of regulation in which SorR1 and SorR2 cooperate at the protein level. While the data are consistent with a model in which SorR1 acts as a transcriptional activator of the pathway, SorR2 appears to act as an inhibitor of the SorR1 activity. This would also explain why there are still low levels of sorbicillinoids detected in the *sorR2* deletion strain while these are completely absent in the *sorR2* overexpression strain in which the pathway is suppressed. This phenotype resembles that of the *aflJ* deletion strain of *Aspergillus*. AflJ and AflR are transcriptional factors that regulate the aflatoxin and sterigmatocystin cluster in *Aspergillus parasiticus* (Chang, 2003; Yu and Keller, 2005). Deletion of the individual genes abolished the production of these compounds. AflR is a transcriptional activator. Like the Δ*sorR2* strain in our study, the aflatoxin structural genes in the *aflJ* deletion strain also remain expressed at low levels, and it has been suggested that AlfJ forms an active complex with AlfR, the main regulator of the pathway (Georgianna and Payne, 2009). Summarizing our results suggests that both transcriptional factors SorR1 and SorR2 orchestrate the biosynthesis of sorbicillinoids, with SorR1 as main transcriptional activator and SorR2 as a repressor of this biosynthetic pathway (Fig. 4B). A similar regulatory mechanism involving two transcriptional factors has been reported for the homologous cluster in *T. reesei* (Derntl *et al*., 2016).

A further observation is that mutational loss of sorbicillinoid production is accompanied by a dramatic reduction in the expression of the pathway genes. A possible explanation of this observation is that sorbicillinoids function as autoinducers. Indeed, when the strain deficient in sorbicillinoid production was fed with filtered medium of a sorbicillinoid‐producing strain, a major upregulation of the sorbicillin gene cluster was noted (Fig. 3). Neither the Δ*sorR1* nor the Δ*sorR2* mutants did show this sorbicillinoid‐dependent transcriptional response. Interestingly, the transcriptional response of the core sorbicillin genes (*sorA, sorB, sorC*) in the *sorR2* deletion strain (Fig. 3B) is similar to what is observed when strain DS68530 is fed with sorbicillinoids (Fig. 3A). A possible scenario that explains these observations is that sorbicillinoids act on SorR2, thereby relieving the inhibitory action of SorR2 on the transcriptional activator SorR1. In this respect, the expression of the sorbicillin cluster expression was partially rescued when the OEsorR2 strain was fed with sorbicillinoids (Fig. 3D). Also, overexpression of SorR1 partially restored transcription, and according to our model, the higher levels of SorR1 overcome the inhibitory effect of SorR2 on transcription. We propose that SorR2 interacts with SorR1 to reduce its transcriptional activating activity, while sorbicillinoids relieve this inhibition by acting on SorR2 (Fig. 4B). This is one of the rare reported examples wherein the product of the synthesis pathway acts as an autoinducer of the expression of the pathway genes. The zearalenone (ZEA) biosynthetic cluster gene of *Fusarium graminearum*, whose regulator isoforms (ZEB2S and ZEB2L) are induced by its own toxin, is another example of this phenomenon (Park *et al*., 2015).

*In silico* analysis indicates that the intergenic DNA region between *sorB* and *sorA* comprises three nucleotide‐binding motifs 5′CGGN_(9)_CGG, which may act as binding sites for SorR1 to regulate the cluster. SorR1 belongs to the family of sequence‐specific DNA‐binding Zn_2_‐Cys_6_ proteins. Such regulators appear to be present in approximately 90% of the PKS‐encoding gene cluster in fungi (Brakhage, 2012). In *Aspergillus flavus*, the deletion of the *aflR* gene that encodes for a Zn_2_‐Cys_6_‐type protein abolished the expression of the aflatoxin and sterigmatocystin cluster, while the overexpression of the same gene increased the expression and production of these secondary metabolites (Yin and Keller, 2011; Brakhage, 2012). SorR1 appears to function in a similar manner. Additionally, our results suggest that there is possible crosstalk between the sorbicillinoid gene cluster regulators (SorR1 and SorR2) and other biosynthetic pathways. When the *sorR1* gene was deleted, enhanced production of compound [13] was observed, whereas in the SorR1 overexpression strain, production of this compound was reduced. Remarkably, this secondary metabolite is not related to sorbicillinoids and was also detected in the *ΔsorA and ΔsorB* strains in which sorbicillinoid biosynthesis is eliminated. Possible crosstalk has been reported before in *Aspergillus nidulans* where the induction of the silent asperfuranone gene cluster was achieved through expression of the *scpR* gene that encodes a transcriptional regulator of the *inp* gene cluster (Bergmann *et al*., 2010; Fischer *et al*., 2016). However, we cannot exclude the possibility that the elevated levels of compound [13] are due to a greater availability of precursor molecules not used for sorbicillinoid biosynthesis.

In conclusion, our results support a model for sorbicillinoid production that includes the functions of the various gene products that are part of the sorbicillinoid gene cluster and an alternative branched path independently of *SorC*. Additionally, it was demonstrated that the regulation of this pathway involves two transcriptional regulators while sorbicillinoids act as autoinducers of this pathway. This work opens possibilities to engineer the sorbicillinoid pathway for the efficient production of novel derivatives of pharmaceutical value.

## Experimental procedures

### Strains, media and growth conditions

*Penicillium chrysogenum* DS68530 was kindly provided by DSM Sinochem Pharmaceuticals (Delft, the Netherlands). All gene deletion and overexpression strains were derived from DS68530Res13 (Sorb407) described by Salo *et al*. (2016); which is a derivative of DS68530. The overexpression strains used in the feed experiments were derived of DS68530 (Table S1). Conidiospores immobilized on rice were inoculated in YGG medium for 48 h to produce fungal protoplasts or for gDNA extraction and for 24 h to produce young mycelium used as pre‐culture inoculum for producing the fermentations. After pre‐culture, the inoculum was diluted seven times in SMP medium (secondary metabolite production medium (Ali *et al*., 2013)) and cells were grown for up to 5 days in shaken flasks at 25 °C and 200 rpm. After 3 and 5 days, samples of the culture medium were collected for RNA extraction and metabolite profile analysis. When indicated, phleomycin agar medium (Snoek *et al*., 2009) supplemented with 60 μg ml^−1^ phleomycin was used for selection and strain purification. Selected transformants were placed on R‐agar for sporulation during 5 days, whereupon the conidiospores were used to prepare rice batches for long‐term storage (Kovalchuk *et al*., 2012).

### Construction of gene deletion and overexpression strains

Gene deletion and overexpression mutants were built using the Gateway Technology (Invitrogen, USA). For creation of deletion strains, 5′ and 3′ regions of each target genes (*Pc21 g05050 (sorR1), Pc21 g05060 (sorC), Pc21 g05070 (sorB), Pc21 g05080 (sorA), Pc21 g05090 (sorR2), Pc21 g05100 (sorT) and Pc21 g05110 (sorD)*) were amplified from gDNA of strain DS68530. All primers used in this study are listed in Table S2. The resistance marker gene (*ble*) for phleomycin was amplified from pJAK‐109 (Pohl *et al*., 2016). Phusion HF polymerase (Thermo Fisher Scientific, USA) was used to amplify all the DNA parts used. The *ble* gene was placed under control of pcbC promoter of *P. chrysogenum*. All the fragments generated were cloned in the respective donor vectors PDONR P4‐P1R, pDONR2R‐P3 and pDONR 221 using BP Clonase II enzyme mix (Invitrogen) and used to transform *E. coli* DH5α, where plasmids were selected for with kanamycin. Next, the constructs were used in an *in vitro* recombination reaction with the pDEST R4‐R3 vector employing LR Clonase II Plus enzyme mix (Invitrogen). Following transformation to *E. coli* DH5α, correct constructs were selected for with ampicillin.

The donor vectors containing the 5′ flank were used to generate the 5′ flanks in the overexpression cassettes. The 3′ flank was generated from amplified homologous regions that were located before and after the start codon of each gene (*sorR1; sorR2*). The *pcbC* (isopenicillin N synthase) gene promoter of *P. chrysogenum* was used to induce expression and was inserted between the two flanks selected. To build the DNA fragment that contains the phleomycin‐resistant cassette, the *ble* gene was amplified from plasmid pFP‐phleo‐122 (Polli *et al*., unpublished) F. Polli, R.A.L. Bovenberg, and A.J.M. Driessen, unpublished data and the *pcbC* promoter which was ordered as a synthetic gene (gBlock) (IDT, USA). The *ble* gene in the phleomycin‐resistant cassette (promoter, gene and terminator) is under control of the *gndA* (6‐phospho‐gluconate dehydrogenase) promoter of *Aspergillus nidulans* (Polli *et al*., 2016). Next, the two fragments were fused by overlap PCR, as described by Nelson and Fitch (2011).

### Fungal transformation

Protoplasts were isolated from *P. chrysogenum* as described previously (Kovalchuk *et al*., 2012). For all transformations, 5 μg of plasmid DNA was linearized with a suitable restriction enzyme, whereupon transformation was performed as described by Weber *et al*. (Weber *et al*., 2012). Screening of transformants was performed by colony PCR using the Phire Plant Direct PCR Kit (Life Technologies, USA). Selected transformants were purified through three rounds of sporulation on R‐agar medium. Transformants were further validated by sequencing integration regions amplified from gDNA.

### Southern blot analysis

A DNA fragment between 0.7 and 0.1 kb from the upstream or downstream region of every gene was amplified and used as a probe for Southern blot analysis (Fig. S1). The probes were labelled with the HighPrime Kit (Roche Applied Sciences, Almere, the Netherlands). About 15 μg of gDNA, previously digested with suitable restriction enzymes, was separated by electrophoresis on an 0.8% agarose gel. The gel was equilibrated in 20× saline–sodium citrate (SSC) buffer (3 M NaCl; 0.3 M C_6_H_5_Na_3_O_7_; pH 7) and the DNA was transferred overnight to a positively charged nylon membrane (Zeta‐Probe; Bio‐Rad, Munchen, Germany). Subsequently, the membrane was incubated overnight with the labelled probe(s). For detection, the membrane was treated with anti‐DIG Fab fragment alkaline phosphatase and the CDP‐Star chemiluminescent substrate (Roche Applied Sciences). The signal was measured using a Lumi‐Imager (Fujifilm LAS‐4000, Fujifilm Co. Ltd, Tokio, Japan).

### qPCR analysis

Mycelium of strains grown for 3 and 5 days in SMP medium was harvested and disrupted in a FastPrep FP120 system (Qbiogene, Cedex, France) to isolate total RNA. The extraction was performed with the TRIzol (Invitrogen) method, and the total RNA obtained was purified using the Turbo DNA‐free kit (Ambion, Carlsbad, CA, USA). RNA integrity was checked on a 2% agarose gel, and the RNA concentration was measured using a NanoDrop ND‐1000 device (ISOGEN, Utrecht, the Netherlands). To synthesize cDNA, 500 ng of RNA was used per reaction using iScript cDNA synthesis kit (Bio‐Rad). The primers used were described previously (Salo *et al*., 2016). The γ‐actin gene (*Pc20 g11630*) was used as a control for normalization (Nijland *et al*., 2010). The SensiMIx SYBR Hi‐ROX (Bioline, Australia) was used as master mix for the qPCR in a MiniOpticon system (Bio‐Rad). The following thermocycler conditions were employed: 95 °C for 10 min, followed by 40 cycles of 95 °C for 15 s, 60 °C for 30 s and 72 °C for 30 s. Measurements were analysed using the Bio‐Rad CFX manager program in which the Ct (threshold cycles) values were determined by regression. To determine the specificity of the qPCRs, melting curves were generated. The analysis of the relative gene expression was performed with $2^{-\Delta \Delta C_{T}}$ method (Livak and Schmittgen, 2001). The expression analysis was performed for two biological samples with at least two technical replicates.

### Metabolite analysis

Strains were grown in SMP medium, and supernatant was collected after 3 and 5 days. Samples were centrifuged for 5 min at 14 000 rpm to remove the mycelium, whereupon 1 mL of the supernatant fraction was filtered with a 2 μm pore polytetrafluoroethylene (PTFE) syringe filter and stored at −80 °C. LC‐MS analysis was performed as described previously (Salo *et al*., 2016). Metabolite analysis was performed with two biological samples with two technical duplicates.

### Other methods

For the feeding experiments with sorbicillinoids, the parental strain DS68530 and its derivatives *ΔsorR1*,* ΔsorR2*, OEsorR1_68530 and OEsorR2_68530 were grown in YGG medium. After 24 h, the inoculum of 3 mL was transferred into a 100 mL shake flask, supplemented with 20 mL of fresh SMP and 2 mL of filtered supernatant that was obtained from a 3‐day‐old culture of the sorbicillinoid‐producing strain DS68530Res13, also grown in SMP. Controls received supernatant of the DS68530 strain, a non‐sorbicillinoid producer. Samples for expression and metabolite analysis were taken at days 1, 3 and 5 of growth.

## Conflict of interest

None declared.

## Author contributions

FGC designed the study, performed the experiments, wrote the manuscript and carried out the data analysis. AJMD conceived the study, supervised and coordinated the design, interpreted the data and corrected the manuscript. YN participated in data analysis and helped to draft the manuscript. PPL and OS supported the mass spectrometry and structural analysis and helped to draft the manuscript. RALB contributed to the coordination of the project and the revision of the manuscript.

## Supporting information


**Fig. S1.** Southern blot analysis of *P. chrysogenum* strains with individual sor gene deletions and *sorR1* and *sorR2* overexpression.
**Table S1**. Strains used in this study.
**Table S2**. Primers used in this study.
