# Supplementary material for: Mechanism and regulation of sorbicillin biosynthesis by Penicillium chrysogenum
Source: Microb Biotechnol. 2017 Jun 15;10(4):958–68. doi: 10.1111/1751-7915.12736 (PMC5481523; doi:10.1111/1751-7915.12736)
Supplement: Supplementary file 1 — Fig. S1. Southern blot analysis of P. chrysogenum strains with individual sor gene deletions and sorR1 and sorR2 overexpression. Table S1. Strains used in this study. Table S2. Primers used in this study. [file MBT2-10-958-s001.docx]

# **Supporting information**

**Mechanism and Regulation of Sorbicillin Biosynthesis by *Penicillium chrysogenum***

Fernando Guzmán-Chávez^1^, Oleksandr Salo^1^, Yvonne Nygård^1,#^, Peter P. Lankhorst^3^, Roel A.L. Bovenberg^2,3^, Arnold J.M. Driessen^1,^*

*Correspondence to Arnold J.M. Driessen

[a.j.m.driessen@rug.nl](mailto:a.j.m.driessen@rug.nl)

| Strain | Genotype | Source |
| --- | --- | --- |
| DS68530 | Penicillin Cluster free, *Δku70*, | DSM Sinochem Pharmaceuticals |
|  | | |
| Strains derived from DS68530: | | |
| DS68530Res13 | AmdS marker, SorA (F146L) | (Salo et al., 2016) |
| *OEsorR1_68530* | Phleo, *pcbC::Pc21g05050* | This study |
| *OEsorR2_68530* | Phleo, *pcbC::Pc21g05090* | This study |
| Strains derived from DS68530Res13: | | |
| *ΔsorR1* | Phleo, *ΔPc21g05050* | This study |
| *ΔsorC* | Phleo, *ΔPc21g05060* | This study |
| *ΔsorB* | Phleo, *ΔPc21g05070* | This study |
| *ΔsorA* | Phleo, *ΔPc21g05080* | This study |
| *ΔsorR2* | Phleo, *ΔPc21g05090* | This study |
| *ΔsorT* | Phleo, *ΔPc21g05100* | This study |
| *ΔsorD* | Phleo, *ΔPc21g05110* | This study |
| *OEsorR1* | Phleo, *pcbC::Pc21g05050* | This study |
| *OEsorR2* | Phleo, *pcbC::Pc21g05090* | This study |
|  |  |  |

Phleo, Phleomycin marker

**Supplementary table 1.** Strains used in this study

|  | Name | Primer sequence (5’→3’) | Reference |
| --- | --- | --- | --- |
| Cloning, gene inactivation | | |  |
| 1 | attB4F_sorR1 | **GGGGACAACTTTGTATAGAAAAGTTG**GTATCAATGGGATGGAATTCCTGAGAGC | This study |
| 2 | attB1R_sorR1 | **GGGGACTGCTTTTTTGTACAAACTTG**GAAGTGTGCGAGGGTTAGTCGATTGC | This study |
| 3 | attB2F_sorR1 | **GGGGACAGCTTTCTTGTACAAAGTGG**GCACGGATAGCAACTGAAGTGACGG | This study |
| 4 | attB3R_sorR1 | **GGGGACAACTTTGTATAATAAAGTTG**CGTCCTTCAAAGCTTTACCAATGTGGC | This study |
| 5 | attB4F_sorC | **GGGGACAACTTTGTATAGAAAAGTTG**GCGAGTTCTTACGCAAGC | This study |
| 6 | attB1R_sorC | **GGGGACTGCTTTTTTGTACAAACTTG**TGCTGGTATGGTAAGCGAC | This study |
| 7 | attB2F_sorC | **GGGGACAGCTTTCTTGTACAAAGTGG**GTGGATTTGAGGAATAACGAC | This study |
| 8 | attB3R_sorC | **GGGGACAACTTTGTATAATAAAGTTG**GAGACTGACTCCTCCAAAGC | This study |
| 9 | attB4F_sorB | **GGGGACAACTTTGTATAGAAAAGTTG**GGCACCATACCACGGTATCC | This study |
| 10 | attB1R_sorB | **GGGGACTGCTTTTTTGTACAAACTTG**CAGCATATCTAACTCATCACC | This study |
| 11 | attB2F_sorB | **GGGGACAGCTTTCTTGTACAAAGTGG**CGTCGGCCGTATTGCCAGACTGC | This study |
| 12 | attB3R_sorB | **GGGGACAACTTTGTATAATAAAGTTG**GCCGCTGTTTCACCCGAGTAACC | This study |
| 13 | attB4F_sorA | **GGGGACAACTTTGTATAGAAAAGTTG**CGTCGGCCGTATTGCCAGACTGC | (Salo et al., 2016) |
| 14 | attB1R_sorA | **GGGGACTGCTTTTTTGTACAAACTTG**GCCGCTGTTTCACCCGAGTAACC | (Salo et al., 2016) |
| 15 | attB2F_sorA | **GGGGACAGCTTTCTTGTACAAAGTGG**GGTCATGTCCGAGAAGCTGTC | (Salo et al., 2016) |
| 16 | attB3R_sorA | **GGGGACAACTTTGTATAATAAAGTTG**CGCCCTTGTTGAAAGGCTCC | (Salo et al., 2016) |
| 17 | attB4F_sorR2 | **GGGGACAACTTTGTATAGAAAAGTTG**CTTGTCCTTCTCTGTAGTAGTAGCAGCAGC | This study |
| 18 | attB1R_sorR2 | **GGGGACTGCTTTTTTGTACAAACTTG**GTGTCAACCAATGAAATAGCAGTCCGTC | This study |
| 19 | attB2F_sorR2 | **GGGGACAGCTTTCTTGTACAAAGTGG**GGACTTTGGAGAAGGGTTGGTTTAGTGG | This study |
| 20 | attB3R_sorR2 | **GGGGACAACTTTGTATAATAAAGTTG**GGCACCTGGAACCTGCACAACC | This study |
| 21 | attB4F_sorT | **GGGGACAACTTTGTATAGAAAAGTTG**GATGCATCTACCTGAGGTCA | This study |
| 22 | attB1R_sorT | **GGGGACTGCTTTTTTGTACAAACTTG**CTGTATTGACTATGGACAAGGC | This study |
| 23 | attB2F_sorT | **GGGGACAGCTTTCTTGTACAAAGTGG**CTGAGTGAGAGCTGTTAGAAATG | This study |
| 24 | attB3R_sorT | **GGGGACAACTTTGTATAATAAAGTTG**ATGACTGCCAAGTCAAGAATAC | This study |
| 25 | attB4F_sorD | **GGGGACAACTTTGTATAGAAAAGTTG**GCGTCAGTTTACATGGCTAT | This study |
| 26 | attB1R_sorD | **GGGGACTGCTTTTTTGTACAAACTTG**GCCTGCATTTTGAGATTG | This study |
| 27 | attB2F_sorD | **GGGGACAGCTTTCTTGTACAAAGTGG**TGAGCGAAGTTCTGACTAGTG | This study |
| 28 | attB3R_sorD | **GGGGACAACTTTGTATAATAAAGTTG**GTACAGATGCAGAGTTGACG | This study |
| Cloning, overexpression | | |  |
| 29 | attB1F_Phleo | **GGGGACAAGTTTGTACAAAAAAGCAGGCTCC**GTCGACTACATGTATCTGCATG | This study |
| 30 | attB2R_Phleo | **GGGGACCACTTTGTACAAGAAAGCTGGGTC**GCAAATTAAAGCCTTCGAG | This study |
| 31 | attB1F_pE+Phleo | **GGGGACAAGTTTGTACAAAAAAGCAGGCTCC**TCTTGCGTTACGGGCGTA | This study |
| 32 | attB2R_Phleo+pIPNS | **GGGGACCACTTTGTACAAGAAAGCTGGGTC**GGTGTCTAGAAAAATAATGGTGAAA | This study |
| 33 | Rv_Phleo+pIPNS | CAGACCAATGCAGCAGGCCCAGTATAAGGAGCAAATTAAAGCCTTCGAG | This study |
| 33 | Fw_Phleo+pIPNS | GTTTTGGGACGCTCGAAGGCTTTAATTTGCTCCTTATACTGGGCCTGC | This study |
| 34 | attB2F_OEsorR1 | **GGGGACAGCTTTCTTGTACAAAGTGGCC**ATGAGAAGGCAACAGTCTGG | This study |
| 35 | attB3R_OEsorR1 | **GGGGACAACTTTGTATAATAAAGTTG**ATCGGAAGACGTGTGTTTATC | This study |
| 36 | attB2F_OEsorR2 | **GGGGACAGCTTTCTTGTACAAAGTGGCC**ATGGAAAATGGATGCACTTC | This study |
| 37 | attB3R_OEsorR3 | **GGGGACAACTTTGTATAATAAAGTTG**ATAGAAGAGCGATCACTCGAT | This study |
| Southern blotting | | |  |
| 38 | KOsorR1_SB_F | CGCATGTGAATTACGTTATG | This study |
| 39 | KOsorR1_SB_R | AGCTCTTCCGAGAAAGAGTC | This study |
| 40 | KOsorC_SB_F | GTTGAAGAGCTCTGGCAATAGT | This study |
| 41 | KOsorC_SB_R | GCTTGCGTAAGAACTCGC | This study |
| 42 | KOsorB_SB_F | GTACGGCAAATAGCTTCCA | This study |
| 43 | KOsorB_SB_R | TGGTAGACGCCTTCTGATC | This study |
| 44 | KOsorA_SB_F | CTACACATTACGGCTTGTACC | This study |
| 45 | KOsorA_SB_R | TGCAACTAGATGCATGTCTTC | This study |
| 46 | KOsorR2_SB_R | GAGTTGACACGTCTCATCG | This study |
| 47 | KOsorR2_SB_R | CTGGCATATCCAAGAATTCC | This study |
| 48 | KOsorT_SB_F | GTGCATGTTCTCCTTACAGACT | This study |
| 49 | KOsorT_SB_R | TGAAGCCAAGAGACAGGTC | This study |
| 50 | KOsorD_SB_F | CGTCAACTCTGCATCTGTAC | This study |
| 51 | KOsorD_SB_R | ATACTTCAAGCACAAGGCTC | This study |
| 52 | OEsorR1_SB_F | GATCCAAATCGACGATAGG | This study |
| 53 | OEsorR1_SB_R | ATCCAGTGCGGTACTTCAT | This study |
| 54 | OEsorR2_SB_F | GCCTTGTCCATAGTCAATACAG | This study |
| 55 | OEsorR2_SB_R | AATGAAGATCACGCAGACA | This study |
| 56 | Cpcr_Phleo_F | ACTACATGTATCTGCATGTTGC | This study |
| 57 | Cpcr _Phleo_R | AATGGAAACGACCTGAGC | This study |
| 58 | Cpcr _sorR1_F | GCAGTAAGAGTGCACTGAAAC | This study |
| 60 | Cpcr _sorR1_R | CATTTATGAACGAGCCGAG | This study |
| 61 | Cpcr _sorC_F | GTATGCTGGAGATGCAAGAC | This study |
| 62 | Cpcr _sorC_R | CTAGATGCCTCACAACTCG | This study |
| 63 | Cpcr _sorB_F | GTGAGTACAAATGCGCCT | This study |
| 64 | Cpcr _sorB_R | ATGTGATACACGGTTCGC | This study |
| 65 | Cpcr _sorA_F | CGACTACTCAGGTCAAGAGTG | This study |
| 66 | Cpcr _sorA_R | AGTGTCTGATGCTGATGAATC | This study |
| 67 | Cpcr _sorR2_F | GACGCCATCAATCAATGT | This study |
| 68 | Cpcr _sorR2_R | GGACTCCGATGAGTTGAGT | This study |
| 69 | Cpcr _sorT_F | GCGAGACTAGTTCGATTCTG | This study |
| 70 | Cpcr _sorT_R | CTGCTTTAGTCTGAGCGCT | This study |
| 71 | Cpcr _sorD_F | GATGGCAAGATCTACGTGAG | This study |
| 72 | Cpcr _sorD_R | CTCAAGTGAATGACCGTAGC | This study |

Nucleobase indicated in bold reflect the *att*B sites for the recombination used by the Gateway Technology.


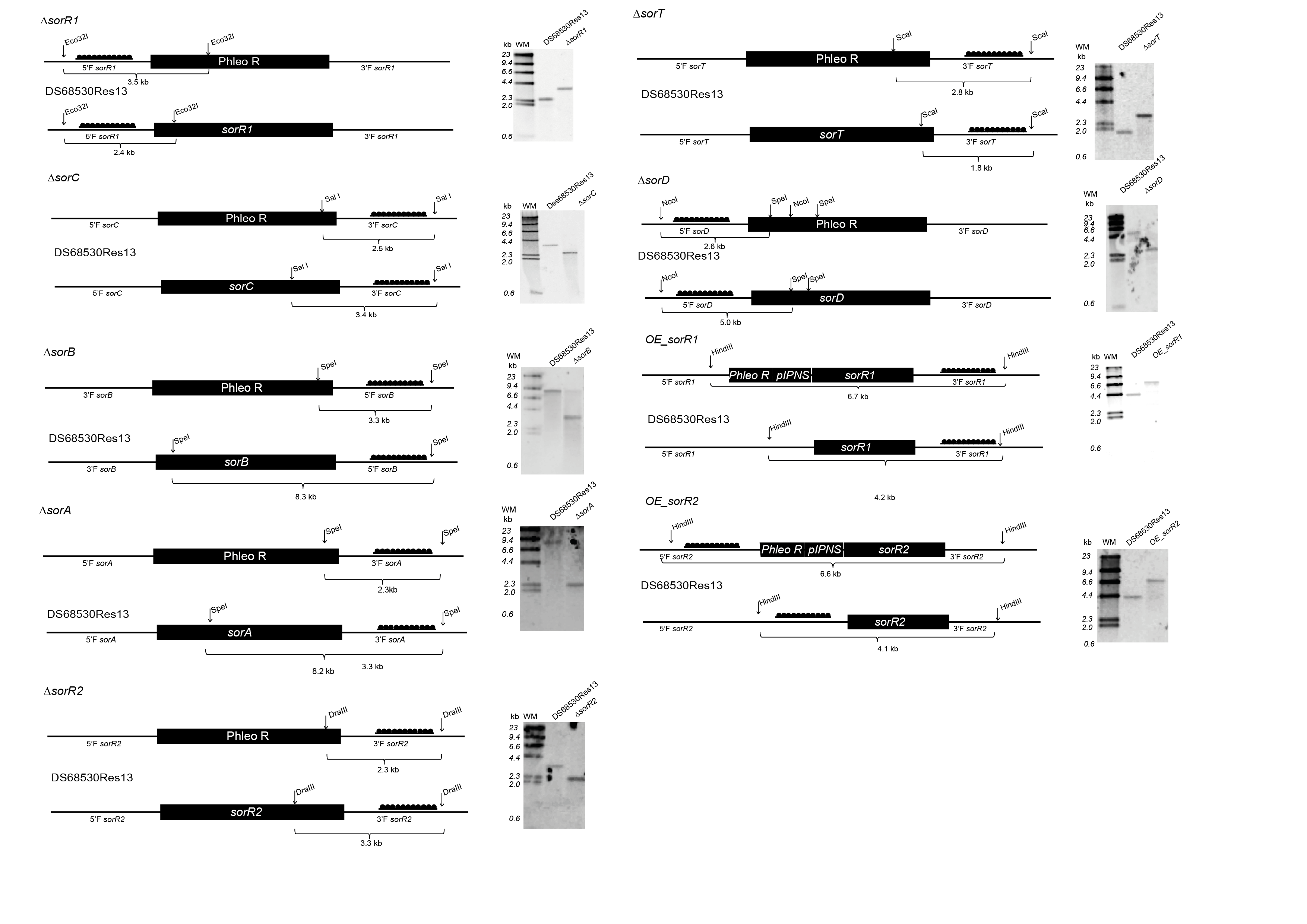
 **Figure S1.** Southern blot analysis of *P. chrysogenum* strains with individual sor gene deletions and *sorR1* and *sorR2* overexpression. gDNA from DS68530Res13 was used as a control. The restriction enzyme used to digest the gDNA and the expected fragment sizes are indicated.
